# Development of cultured *Plasmodium falciparum* blood-stage malaria cell banks for early phase *in vivo* clinical trial assessment of anti-malaria drugs and vaccines

**DOI:** 10.1186/s12936-015-0663-x

**Published:** 2015-04-07

**Authors:** Danielle I Stanisic, Xue Q Liu, Sai Lata De, Michael R Batzloff, Tanya Forbes, Christopher B Davis, Silvana Sekuloski, Marina Chavchich, Wendy Chung, Katharine Trenholme, James S McCarthy, Tao Li, B Kim Lee Sim, Stephen L Hoffman, Michael F Good

**Affiliations:** Institute for Glycomics, Griffith University, Southport, QLD Australia; Clinical Tropical Medicine Laboratory, QIMR Berghofer Medical Research Institute, University of Queensland, Herston, Australia; Australian Army Malaria Institute, Enoggera, QLD Australia; Sanaria Inc, Rockville, MD USA

**Keywords:** Malaria, *Plasmodium falciparum*, Cell bank, Good Manufacturing Practices

## Abstract

**Background:**

The ability to undertake controlled human malaria infection (CHMI) studies for preliminary evaluation of malaria vaccine candidates and anti-malaria drug efficacy has been limited by the need for access to sporozoite infected mosquitoes, aseptic, purified, cryopreserved sporozoites or blood-stage malaria parasites derived *ex vivo* from malaria infected individuals. Three different strategies are described for the manufacture of clinical grade cultured malaria cell banks suitable for use in CHMI studies.

**Methods:**

Good Manufacturing Practices (GMP)-grade *Plasmodium falciparum* NF54, clinically isolated 3D7, and research-grade *P. falciparum* 7G8 blood-stage malaria parasites were cultured separately in GMP-compliant facilities using screened blood components and then cryopreserved to produce three *P. falciparum* blood-stage malaria cell banks. These cell banks were evaluated according to specific criteria (parasitaemia, identity, viability, sterility, presence of endotoxin, presence of mycoplasma or other viral agents and *in vitro* anti-malarial drug sensitivity of the cell bank malaria parasites) to ensure they met the criteria to permit product release according to GMP requirements.

**Results:**

The *P. falciparum* NF54, 3D7 and 7G8 cell banks consisted of >78% ring stage parasites with a ring stage parasitaemia of >1.4%. Parasites were viable *in vitro* following thawing. The cell banks were free from contamination with bacteria, mycoplasma and a broad panel of viruses. The *P. falciparum* NF54, 3D7 and 7G8 parasites exhibited differential anti-malarial drug susceptibilities. The *P. falciparum* NF54 and 3D7 parasites were susceptible to all anti-malaria compounds tested, whereas the *P. falciparum* 7G8 parasites were resistant/had decreased susceptibility to four compounds. Following testing, all defined release criteria were met and the *P. falciparum* cell banks were deemed suitable for release. Ethical approval has been obtained for administration to human volunteers.

**Conclusions:**

The production of cultured *P. falciparum* blood-stage malaria cell banks represents a suitable approach for the generation of material suitable for CHMI studies. A key feature of this culture-based approach is the ability to take research-grade material through to a product suitable for administration in clinical trials.

**Electronic supplementary material:**

The online version of this article (doi:10.1186/s12936-015-0663-x) contains supplementary material, which is available to authorized users.

## Background

Significant advances have been made in the control of malaria, yet half of the world’s population remains at risk. In 2012, there were over 200 million cases of malaria and 627,000 malaria-attributable deaths, highlighting the need for continued development of malaria control strategies [1]. Sustainable malaria vaccine and drug development requires access to reliable systems, such as controlled human malaria infections (CHMIs), in which to obtain preliminary human efficacy data to enable the selection of candidates for further evaluation in malaria endemic countries. Additionally, these systems can facilitate the investigation of immunological responses that are induced in response to malaria infection [2-4].

CHMI designed to assess vaccine candidates and anti-malaria drugs in malaria-naïve volunteers have predominantly utilized experimental malaria infections induced either by feeding laboratory-reared *Plasmodium falciparum*-infected mosquitoes on study participants [5-8] or injecting erythrocytes containing blood-stage malaria parasites [3,9-11]. Recently, cryopreserved sporozoites administered intradermally and intramuscularly, have also been successfully used to initiate infection [12-14]. While these CHMI approaches are routinely used at a number of centres in the USA, Netherlands, UK and Australia, the ability to implement, maintain and use them is limited in part by access to suitable material and this has been restricted by technical considerations including access to insectary facilities to house and feed mosquitoes and to *Plasmodium*-infected individuals.

Previous studies that have utilized a cell bank of *Plasmodium*-infected erythrocytes to induce experimental malaria infections have used parasitized erythrocytes that were cryopreserved directly *ex vivo* from a parasitaemic volunteer who had been deliberately inoculated by mosquitoes infected with *P. falciparum* 3D7 [15]. This material has been used in numerous studies with diverse endpoints and has been administered to >200 volunteers [3,9,11]. More recently, a malaria cell bank was collected from a returned traveller infected with *P. vivax* and has been used to infect human volunteers [16]. These studies, together with decades of experience with blood transfusion and significant advances in the testing utilized for detection of infection of donor blood with blood-borne pathogens have helped to overcome many of the ethical and safety concerns that were deemed to be associated with administering *Plasmodium*-infected erythrocytes for CHMI [2].

While these cell banking methods represent obvious ways to obtain suitable material for blood-stage CHMIs, they rely on access to deliberately infected volunteers/malaria-infected returned travellers and ethical approval to collect, store and use this material. An *ex vivo* cell bank is also a finite resource which will eventually be exhausted. Additionally, if the *Plasmodium*-infected donor is not of the “universal” O Rh negative blood type, then this places additional restrictions on the selection of potential recipients, limiting it to those with a compatible blood type.

An alternative approach for the production of a cell bank of *P. falciparum*-infected erythrocytes is to exploit the relatively straightforward methodology for the continuous *in vitro* culture of malaria parasites (for example [17]) to generate a large quantity of *Plasmodium*-infected erythrocytes that can then be cryopreserved to produce a cell bank. As this material is for clinical use, production must follow current, local GMP regulatory requirements and guidelines for investigational products. This involves implementing a GMP environment suitable for the manufacture of Investigational Medicinal Products (Annex 13 of the Pharmaceutical Inspection Convention and Pharmaceutical Inspection Co-operation Scheme (PIC/S) Guide) which encompasses the establishment of robust quality management systems and standard operating procedures, rigorous documentation to monitor and control the manufacturing process and utilising accredited contract research organizations to undertake the relevant testing of the final product. Administration of this material to human volunteers is also governed by regulatory and ethical guidelines. However, this culture-based approach has numerous advantages, including permitting the selection of the parasite strain and the blood type of the erythrocytes used to propagate the parasite.

Three cultured malaria cell banks, consisting of *Plasmodium falciparum*-infected erythrocytes, have been produced using GMP-grade, clinically isolated or research (non-GMP)-grade parental cell lines as starting material. Described below are the manufacturing process and *in vitro* characterization of these *P. falciparum* blood-stage malaria cell banks that is required to achieve approval for human use.

## Methods

The manufacture and release of three *Plasmodium falciparum* blood-stage malaria cell banks, suitable for use in CHMI studies, was undertaken at The Institute for Glycomics, Griffith University, Australia (*P. falciparum* NF54 and 7G8) and Q-Gen, QIMR Berghofer Medical Research Institute (*P. falciparum* 3D7).

### Malaria parasites

A vial of GMP-grade *P. falciparum* NF54 master cell bank was provided by Sanaria Inc. [18]. This parasite was originally isolated from a returned traveller, resident in Amsterdam [19]. A vial of the same provenance, but isolated from a subject with a sporozoite-induced infection [15] was used as the source of *P. falciparum* 3D7 parasites. A vial of research (non-GMP)-grade *P. falciparum* 7G8 was provided by the Australian Army Malaria Institute, Queensland, Australia. This parasite is a clone, derived from the isolate IMTM 22, which originated in Brazil [20].

### Culture of *P. falciparum* parasites for the production of the blood-stage malaria cell banks

The culturing processes for producing the *P. falciparum* NF54 and 7G8 cell banks were undertaken at Griffith University whereas the production of the *P. falciparum* 3D7 bank was carried out at Q-Gen, QIMR Berghofer Medical Research Institute. All of the processes were carried out in compliance with the Annex 13, PIC/S Guide, in cleanrooms and a monitored environment suitable for production of sterile biologics in accordance with approved protocols.

Leukocyte-depleted O negative erythrocytes used during the manufacturing process were supplied by Key Biologics, LLC, Memphis, TN, USA for the *P. falciparum* NF54 and *P. falciparum* 7G8 cell banks and the Australian Red Cross Blood Service (ARCBS) for the *P. falciparum* 3D7 cell bank. Serum for parasite culture for all three banks was supplied by Key Biologics. All of the materials obtained from blood donors were compliant with the US Federal Drug Administration (FDA) and Australian Therapeutic Goods Administration (TGA) Order 88. The donors of these materials had been screened for infectious agents in compliance with applicable TGA and FDA regulations, requirements and guidelines for blood screening and blood collection. Additionally, donors were asked specific questions to ascertain the possibility of recent exposure to the malaria parasite and anti-malarial antibody testing was carried out on the blood components. Prior to the use of these blood components in the manufacturing process, aliquots of erythrocytes and serum were tested to confirm that they could support the growth of the malaria parasite.

The expansion and production of these blood-stage malaria cell banks was carried out as previously published [15]. Briefly, seed vials containing the *P. falciparum* NF54, 3D7 or 7G8 parasitized red blood cells in Glycerolyte 57 solution (Baxter) were retrieved from liquid nitrogen and taken to the cleanroom for processing. The vial was placed in a water bath at 37°C for 1–2 minutes until thawed. Deglycerolization of the sample was performed as follows. An equal volume of warmed 12% NaCl was added drop-wise while gently shaking the tube. The mixture was allowed to stand for 5 minutes at ambient temperature. Following this, 9x the cell suspension volume of 1.6% NaCl was added drop-wise while gently shaking the tube. The tube was then centrifuged at 433 g for 5 minutes at ambient temperature. Following removal of the supernatant, 9x the original cell suspension volume of 0.9% NaCl was added drop-wise while gently shaking the tube. The tube was centrifuged again at 433 g for 5 minutes at ambient temperature. The cell pellet was measured and human blood group O Rh negative erythrocytes were added so that the total cell pellet was at a 5% haematocrit. This cell pellet was then suspended in 6 ml of RPMI-1640 (Gibco) supplemented with 10% heat inactivated human serum and 0.01 mg/ml gentamicin (Gibco). The cell suspension was transferred to a tissue culture dish and was placed in an Astec multi-gas incubator (5% O_2_, 5% CO_2_ and 90% N_2_) at 37°C. The parasites were then cultured as described previously [17]. Parasite cultures were checked regularly, at which time, thin blood films were made from collected samples, stained with Diff Quik (Bacto Laboratories) and read to ascertain the parasitaemia. As required, the parasites were sub-cultured to 0.5% parasitaemia with freshly washed human erythrocytes to maintain a 5% haematocrit and placed back in the multi-gas incubator. This culturing process was continued with the number of tissue culture dishes/flasks increasing until the malaria parasite was at ring stage and it was calculated that there was adequate material to cryopreserve for the cell bank. Immediately prior to cryopreservation, thin blood films were made to define the parasitaemia of the produced cell banks. Slides were stained with Diff Quik and read 4 times by 2 independent microscopists, counting a minimum of 1,000 cells for each read. The average of these 4 reads was used to define the parasitaemia of the cell bank.

### Cryopreservation of the *P. falciparum* blood-stage malaria cell banks

To cryopreserve the ring stage parasitized erythrocytes for a cell bank, the cell suspensions from the different flasks/dishes were pooled and centrifuged at 433 g for 10 minutes. Following removal of the supernatants, the resulting cell pellets were pooled into a single flask and the total pellet volume measured. Twice the cell pellet volume of Glycerolyte 57 was added drop-wise while gently mixing by shaking the flask. One millilitre of pRBC/Glycerolyte 57 suspension was placed in each labelled cryovial. Cryovials were transported to a Planer Kryo 560–16 Controlled Rate Freezer and were frozen according to the following programme: 1°C decrease/minute until the temperature reached −70°C, 10°C decrease/minute until the temperature reached −180°C. Following completion of the freezing programme, cryovials were transferred to liquid nitrogen for long-term storage. A proportion of the cryovials were either stored separately as retention samples to allow for future retrospective analysis, if required or were allocated for release testing of the cell banks according to specific criteria (see Additional file 1).

### Assessment of *P. falciparum* blood-stage malaria cell bank parasite viability after thawing by tritiated hypoxanthine uptake

Incorporation of ^3^[H]-hypoxanthine was performed as previously described [21] with some modifications. Briefly, a vial of the *P. falciparum* NF54 and 7G8 cell banks was thawed, as outlined above, and immediately added in quadruplicate to a 96-well flat bottom plate at 2% haematocrit in RPMI-1640 (Gibco) supplemented with 10% heat inactivated human serum and 0.01 mg/ml gentamicin (Gibco). Non-parasitized human erythrocytes were added to the plate as a control. Parasitized and non-parasitized erythrocytes were incubated in the presence of 0.5 μCi/well of ^3^[H]-hypoxanthine (Amersham Pharmacia) for up to 36 hours. Plates were then placed at −80°C overnight. Following thawing, cells were harvested onto glass fibre mats (Perkin Elmer) using a Filtermate cell harvester (Perkin Elmer) and ^3^[H]-hypoxanthine incorporation was determined using a Microbeta^2^ Microplate Counter (Perkin Elmer).

### Assessment of the *in vitro* cloning efficiency of the *P. falciparum* blood-stage malaria cell bank parasites by PfHRPII production

Quantitative viability of the *P. falciparum* NF54 and 7G8 cell bank parasites was determined by measuring the cloning efficiency via a limit dilution approach with positivity determined by production of PfHRPII. This was undertaken using the SD Malaria Antigen P.f ELISA kit (Standard Diagnostics), a sandwich enzyme-linked immunosorbent assay for the qualitative detection of *P. falciparum* histidine rich protein II (HRP-II).

A vial of the *P. falciparum* NF54 and 7G8 cell banks were thawed, as outlined above. Cell pellets were re-suspended in RPMI-1640 (Gibco) supplemented with 10% heat inactivated human serum and 0.01 mg/ml gentamicin (Gibco). Based on the recorded parasitaemia of the cell bank when it was cryopreserved, the cell bank parasites were plated out in 96-well flat bottom plates at 2% haematocrit at theoretical concentrations of 40, 4, 2, 1, 0.5 parasites/well with 100 μl of parasite suspension added/well. Each concentration was plated out in replicates of 30 and non-parasitized erythrocytes were included as a negative control. Plates were placed in an atmosphere of 90% N_2,_ 5% O_2_, 5% CO_2_, at 37°C for 7 days. On Day 4, the media was removed from each well and replaced with fresh media. On Day 7, plates were processed for detection of *P. falciparum* HRPII. The manufacturer’s instructions were followed, with minor modifications. Following mixing, 50 μl of the parasite suspension from each well was transferred to the corresponding well of an uncoated microplate. Seventy-five microlitres of the working enzyme conjugate (anti-mouse *Plasmodium falciparum* HRPII Ig conjugated to horseradish peroxidase in lysis buffer) was then added into each well of the uncoated microplate. Plates were incubated at ambient temperature for 30 minutes. One hundred microlitres was then transferred from each well of the uncoated microplate to the corresponding well of the pre-coated microplate. Plates were incubated at 37°C for 90 minutes. Following this, plates were washed 6 times with the wash buffer and 100 μl of tetramethylbenzidine substrate was added to each well. Plates were incubated at ambient temperature for 10 minutes, after which 100 μl of a 1 N Hydrochloric acid stop solution was added to stop the reaction. Plates were read on an xMark microplate spectrophotometer (Biorad) at 450 nm, with a reference wavelength of 620 nM. Detection of *P. falciparum* HRPII was used to define a parasite positive well, with the cut-off for positivity calculated as the mean + 3 standard deviations of the wells containing normal erythrocytes alone. A limiting dilution analysis was undertaken to determine the frequency of viable parasites [22].

### Sterility testing of *P. falciparum* blood-stage malaria cell banks

Sterility testing for the assessment of bio-contamination with aerobic and anaerobic microorganisms was undertaken. For *P. falciparum* NF54 and 7G8, this was performed by Biotest Laboratories Pty Ltd (Underwood, Australia) using the direct inoculation technique into tryptone soya broth and thioglycollate medium. For *P. falciparum* 3D7, the BacT/ALERT® system (bioMerieux) was employed, using a process that has been validated by the ARCBS for detection of microorganisms in human blood products. Test parameters and acceptance criteria were defined according to the British Pharmacopoeia 2014, Appendix XVI A.

### Mycoplasma testing of *P. falciparum* blood-stage malaria cell banks

Mycoplasma testing was undertaken by ams Laboratories Pty Ltd (Silverwater, Australia) (for *P. falciparum* NF54 and 7G8) or Q-Gen, QIMR Berghofer Medical Research Institute (*P. falciparum* 3D7), using a PCR-based method. The limit of sensitivity of the assay was 10 copies/ml.

### Endotoxin testing of *P. falciparum* blood-stage malaria cell banks

Endotoxin testing was undertaken by ams Laboratories Pty Ltd (Silverwater, Australia) (for *P. falciparum* NF54 and 7G8) using a Kinetic Chromogenic LAL test and Biotest Laboratories (Underwood, Australia) (for *P. falciparum* 3D7) in accordance with the British Pharmacopoeia Appendix XIV C. The detection limit of the assay was 0.5 or 0.06 Endotoxin Units (EU)/ml respectively.

### Viral testing of the *P. falciparum* blood-stage malaria cell banks

Viral testing of the initial *P. falciparum* NF54 master cell bank vial provided by Sanaria was performed in compliance with applicable FDA guidelines. As it was provided as a GMP-grade parental cell line, and blood components collected from screened donors were used in the production of the cell bank, the Griffith University *P. falciparum* NF54 cell bank was not re-tested.

Viral testing of the cultured *P. falciparum* 7G8 bank was performed by Charles River Pharmaceutical Services GmbH (Germany) and was undertaken in compliance with applicable TGA guidelines. RT-PCR assays for HAV (Hepatitis A virus), HBV (Hepatitis B virus), HCV (Hepatitis C virus), HHV-6 (Human Herpes Virus 6), HHV-7 (Human Herpes Virus 7), HHV-8 (Human Herpes Virus 8), HIV-1 (Human Immunodeficiency Virus 1), HIV-2 (Human Immunodeficiency Virus 2), HTLV-I (Human T cell Lymphotropic Virus 1), HTLV-II (Human T cell Lymphotropic Virus 2), SV40 (Simian virus 40), EBV (Epstein Barr Virus), CMV (Cytomegalovirus) and Parvovirus B19 and PCR assays for *Treponema paraluiscuniculi* and *T. palladium* were performed.

The clinically isolated *P. falciparum* 3D7 parental cell bank was derived from blood collected from a donor that had been infected with malaria through the bites of infected mosquitoes under controlled conditions [15]. The donor was screened extensively for blood borne viruses prior to infection and then re-screened 12 months following blood collection and had no evidence of blood borne infections at these time points. Moreover, this *P. falciparum* 3D7 parental cell bank has been directly inoculated into >200 human volunteers with pre-and post-inoculation testing not showing any evidence of seroconversion to adventitious viruses. The blood components used in the culturing process were also obtained from screened donors. Therefore, the risk for presence of blood borne viruses in the *P. falciparum* 3D7 bank was deemed to be minimal, and it was not retested.

### Identity testing of the *P. falciparum* cell bank parasites

Identity testing was performed by Sanaria on the original Sanaria GMP-grade *P. falciparum* NF54 cell bank that was used as the starting material for the Griffith University cell bank. As this was provided as a GMP-grade starting seed vial, further identity testing of the Griffith University *P. falciparum* NF54 cell bank parasites was not undertaken. Identity testing performed by Sanaria was according to published methods and consisted of typing the malaria parasites based on a polymorphic microsatellite (PfRRM) within a known multicopy PfRR repetitive element of *P. falciparum* [23,24]. The resulting band pattern was matched to that of the reference standard isolate.

Identity testing was performed on the clinically isolated *P. falciparum* 3D7 parental cell bank that was used as the starting material for the *P. falciparum* 3D7 bank. It was performed by DNA sequence analysis of three polymorphic genes (*msp1*, *msp2* and *glurp*), which verified sequence identity to the reference *P. falciparum* 3D7 strain. Therefore, identity testing of the *P. falciparum* 3D7 cell bank parasites was not undertaken.

Identity testing of the *P. falciparum* 7G8 cell bank was undertaken by Griffith University in accordance with approved procedures as the starting seed bank material provided was research-grade. An MSP1 genotyping assay was undertaken according to published methods, using the R033 primers for the nested reaction [25]. The MSP1 nested amplicons were sequenced by the Australian Genome Research Facility Ltd and this was evaluated against that of the *P. falciparum* 7G8 reference standard isolate.

### *In vitro* anti-malaria drug susceptibility of the *P. falciparum* blood-stage malaria cell bank parasites

Anti-malaria drug testing was performed on the *P. falciparum* NF54 and 7G8 cell banks due to possible concerns that the drug resistance phenotype of the parasite may have altered following culture. Testing was performed at the Army Malaria Institute, Enoggera, Queensland, Australia. The ten anti-malaria compounds used were: artemether (WWARN), artesunate (WWARN), atovaquone (Sigma), chloroquine (Sigma), dihydroartemisinin (DKPHarm, Vietnam), lumefantrine (WWARN), mefloquine (Sigma), proguanil (Jacobus Pharmaceutical Company, Inc.), piperaquine (WWARN) and pyrimethamine (WRAIR). One hundred percent methanol was used to prepare the stock solution of DHA, 50% methanol was used for artemether, chloroquine and piperaquine, whereas the rest of the compounds were dissolved in DMSO.

*Plasmodium falciparum* 7G8 and NF54 cell bank parasites were placed in continuous culture in RPMI-1640-LPLF (Gibco, Invitrogen Corporation, CA), supplemented with 5.97 g/L HEPES buffer, 2.0 g/L D-glucose, 0.05 g/L hypoxanthine, 40 mg/L gentamycin, 0.21% sodium bicarbonate (added fresh) and containing 10% human plasma and 4% O(+) red blood cells in an atmosphere of 90% N_2,_ 5% O_2_, 5% CO_2_, at 37°C as described previously [17]. *Plasmodium falciparum* D6 was tested in parallel to the *P. falciparum* NF54 cell bank parasites, due to its’ known drug sensitivity profile. Cultures were routinely synchronized using D-sorbitol [21], when the majority of parasites (>85%) were at early trophozoite (ring) stage.

The *in vitro* anti-malaria activities of 10 compounds were assessed using the [^3^H]-hypoxanthine growth inhibition assay [21]. Briefly, synchronized parasite cultures (>90% rings, 4–8 hours post invasion in culture medium lacking hypoxanthine) with a starting parasitaemia of 1% and at 2% haematocrit, were exposed to the compounds at 10 two-fold concentrations in 96-well plates (100 μl per well). Uninfected red blood cells at 2% haematocrit were used as background controls. The plates were incubated in the gas mixture at 37°C. For all but two antifolate compounds, proguanil and pyrimethamine, the assay duration was 48 hours with [^3^H]-hypoxanthine (0.2 μCi/well) added at trophozoite stage approximately 24 hours from the start of the experiment. For proguanil and pyrimethamine assays, the initial parasitaemia was 0.5% and parasite cultures were exposed to the compounds for 96 hours with [^3^H]-hypoxanthine added after the first 48 hours.

Following incubation, plates were frozen, then subsequently thawed and harvested using Tomtech Harvester 96 Mach II and radioactive counts were obtained using Wallac TriLux 1450 Microbeta Liquid Scintillation Counter (Perkin Elmer, USA). All assays were performed in triplicate. For each compound, the data from at least two independent experiments were obtained and analysed using Graph Prism Software (GraphPad Prism V5.0, GraphPad Software, Inc., CA). Drug concentration values were transformed into logarithmic values. After subtracting the background values, the data from drug-treated wells were normalized against drug-free control wells. Non-linear regression analysis was carried out on the log values of the compound’s concentration *vs* parasitic hypoxanthine incorporation to determine inhibitory concentrations. The *in vitro* anti-malarial activity of the compound was defined as inhibitory concentrations (IC_50_) and (IC_90_) that cause 50% and 90% inhibition of parasite growth.

Testing of the *P. falciparum* 3D7 cell bank parasites was performed using similar methodology with minor modifications. The anti-malaria compounds used were: amodiaquine, artemisinin, chloroquine, dihydroartemisinin, mefloquine, pyronaridine, quinine and sulphadoxine.

## Results

### Evaluation of the parasitaemia of the *P. falciparum* cell banks

Thin blood smears that had been prepared from the *P. falciparum* cell banks immediately prior to cryopreservation were examined and read to define the parasite stages present and the parasitaemia of the cell banks. The *P. falciparum* NF54 cell bank contained 80% of parasites at ring stage with a 4.5% ring stage parasitaemia. The *P. falciparum* 7G8 cell bank contained 78% of parasites at ring stage with a 1.4% ring stage parasitaemia. Similarly, the *P. falciparum* 3D7 bank contained 80% of parasites at ring stage with a 3% ring stage parasitaemia.

### Confirmation of the identity of the *P. falciparum* cell bank parasites

The Griffith University *P. falciparum* NF54 cell bank and the *P. falciparum* 3D7 cell bank were not tested to confirm their identity as the parental cell banks, from which they were derived, have a well characterized provenance and were manufactured in a cleanroom environment. Thus, the possibility of cross-contamination with another malaria parasite was considered negligible.

Genotyping was undertaken on the Griffith University *P. falciparum* 7G8 cell bank to confirm its identity, as it was derived from research-grade material. Following a primary amplification of the block 2 region of MSP1, a nested reaction using the R033 allelic type-specific primers was performed. Analysis of the sequence data for the nested amplicon revealed 100% identity between the Griffith University *P. falciparum* 7G8 cell bank parasites and the *P. falciparum* 7G8 reference strain.

### Evaluation of the viability of the *P. falciparum* cell bank parasites

Following thawing of the cell bank vials, the viability of the cell bank parasites was assessed in 3 ways. For all three cell banks, blood smears were prepared from cultures on the day of thawing and days 1–3 following initiation of culture. Growth of the cell bank parasites, indicating viability, was confirmed by regular microscopy of the cultures. Passage of the parasites into subsequent asexual cycles was demonstrated by confirming the appearance of the ring stage parasites in significantly increased numbers.

**Figure 1:**
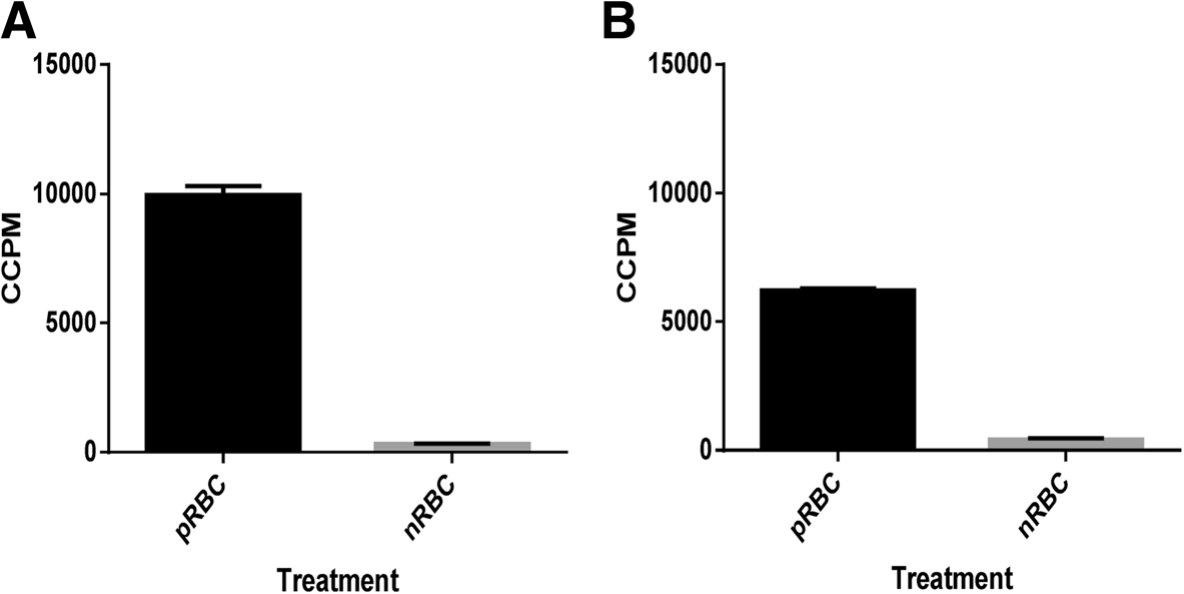
**Uptake of [** ^**3**^ **H]-hypoxanthine by A.** ***P. falciparum*** **NF54 and B.** ***P. falciparum*** **7G8 cell bank parasites.** Each treatment was tested in quadruplicate; columns represent the mean + SE. pRBC: parasitized red blood cells, nRBC: normal red blood cells.

For the *P. falciparum* NF54 and 7G8 cell bank parasites, viability was also assessed by incorporation of [^3^H]-hypoxanthine into newly synthesized parasitic DNA. Radioisotope uptake was demonstrated for the parasites in these *P. falciparum* cell banks, in comparison with the non-parasitized erythrocyte control (Figure 1).

**Figure 2:**
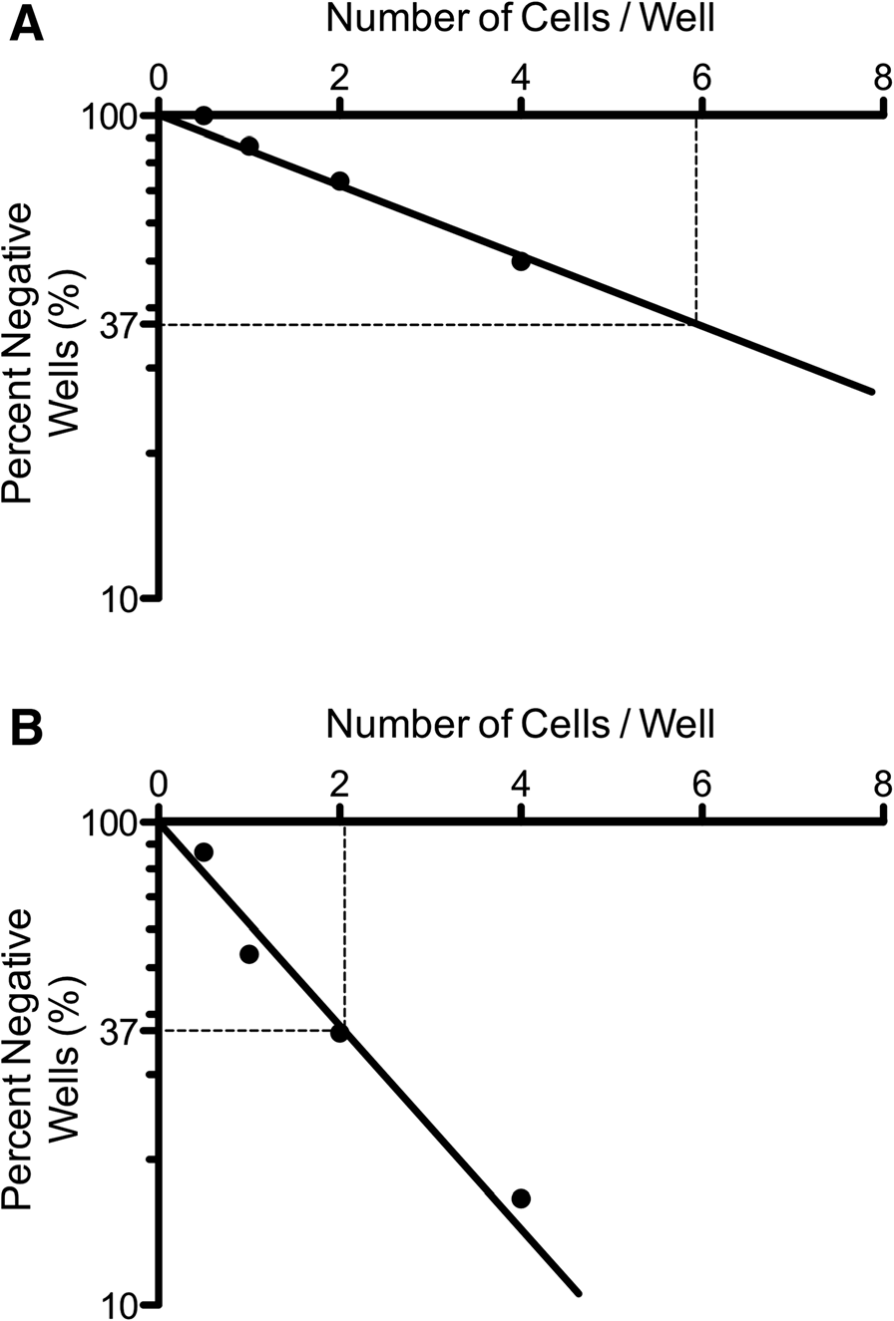
**Graphical representation of limiting dilution analysis results according to a single-hit Poisson model.** Data are from HRPII assays performed on **A**. *P. falciparum* 7G8 and **B**. *P. falciparum* NF54 cell bank parasites. Positivity was determined based on HRPII production.

Finally, for the *P. falciparum* NF54 and 7G8 cell bank parasites, the percentage of viable parasites in the cell banks was derived from a limiting dilution assay [22], in which the frequency of viable, parasitized cells was determined by limit dilution analysis with positivity determined by production of *P. falciparum* HRPII. The cell dose yielding 37% negative wells was determined by interpolation and this was used to estimate the frequency of responding cells in each of the malaria cell banks. Thus, the number of responding cells in the *P. falciparum* 7G8 bank was estimated at approximately 1:6 (Figure 2), equating to an approximate 15% viability of malaria parasites in this bank. The number of responding cells in the *P. falciparum* NF54 cell bank was estimated at approximately 1:2 (Figure 2), which equates to an approximate 50% viability of malaria parasites in this bank.

### Detection of mycoplasma in the *P. falciparum* cell banks

There was no evidence of mycoplasma contamination in any of the three expanded *P. falciparum* cell banks.

### Sterility testing and measurement of endotoxin in the *P. falciparum* cell banks

Following a 14 day incubation period, there was no evidence of growth of aerobic or anaerobic microorganisms. The endotoxin testing revealed <0.500 EU/ml in each of the produced cell banks.

### Testing for viral contaminants in the *P. falciparum* cell banks

Viral testing of the original Sanaria *P. falciparum* NF54 cell bank was undertaken to comply with and satisfy applicable FDA guidelines. The Griffith University *P. falciparum* NF54 cell bank and the P. *falciparum* 3D7 cell bank were not tested as they were derived from defined parental cell banks and were manufactured using blood components that had been screened for infectious agents in compliance with applicable TGA, FDA and ARCBS regulations, requirements and guidelines.

As the *P. falciparum* 7G8 cell bank was derived from research-grade material, viral testing was undertaken to exclude the presence of adventitious agents that may have been present in this starting material and was undertaken to comply with applicable TGA guidelines. Viral testing of the *P. falciparum* 7G8 cell bank demonstrated that it was negative for all agents that were tested for ie HAV, HBV, HCV, HHV-6, HHV-7, HHV-8, HIV-1, HIV-2, HTLV-I, HTLV-II, SV40, EBV, CMV, Human Parvovirus B19, *Treponema paraluiscuniculi* and *T. palladium*.

### Evaluation of the *in vitro* susceptibility of the *P. falciparum* cell bank parasites to anti-malaria compounds

The *in vitro* anti-malaria activities (expressed as IC_50_ and IC_90_ values) of the 10 compounds evaluated against the *P. falciparum* NF54 and 7G8 cell bank parasites are shown in Tables 1 and 2 respectively. *P. falciparum* D6 was tested as a reference strain, in parallel to *P. falciparum* NF54, due to its known drug sensitivity profile [26]. Similar to the drug-sensitive *P. falciparum* D6 line (Additional file 2), *P. falciparum* NF54 cell bank parasites were sensitive to all anti-malaria compounds tested (Table 1). *P. falciparum* 7G8 cell bank parasites were susceptible to all the tested artemisinin derivatives (artemether, artesunate and dihydroartemisinin) as well as to atovaquone, mefloquine and lumefantrine (Table 2). It was resistant to chloroquine, proguanil and pyrimethamine and had a decreased susceptibility to piperaquine. The *P. falciparum* 3D7 cell bank parasites were susceptible to all of the eight anti-malaria compounds that were tested (Additional file 3).

**Table 1:** ***In vitro*** **anti-malarial activities (IC**_**50**_ **and IC**_**90**_ **) of 10 anti-malaria compounds against the** ***P. falciparum*** **NF54 cell bank parasites**

**Compound**	**Maximal concentration tested (nM)**	**IC**_**50**_ **(nM)**		**IC**_**90**_ **(nM)**	
		**Average (n = 2)**	**Standard deviation (n = 2)**	**Average (n = 2)**	**Standard deviation (n = 2)**
Artemether	50	5.9	1.7	9.9	2.4
Artesunate	50	3.3	0.2	6.0	0.4
Dihydroartemisinin	35	2.5	0.1	5.0	0.6
Atovaquone	25	0.22	0.06	1.3	0.5
Mefloquine	728	45	6	85	13
Piperaquine	200	19	6	28	9
Lumefantrine	800	118	14	318	166
Chloroquine	250	11.2	1.4	14.7	2.0
Pyrimethamine	50	0.57	0.04	1.28	0.15
Proguanil	200,000	292	20	1,335	504

**Table 2:** ***In vitro*** **anti-malarial activities (IC**_**50**_ **and IC**_**90**_ **) of 10 anti-malaria compounds against the** ***P. falciparum*** **7G8 cell bank parasites**

**Compound**	**Maximal concentration tested (nM)**	**IC**_**50**_ **(nM)**		**IC**_**90**_ **(nM)**	
		**Average (n = 2)**	**Standard deviation (n = 2)**	**Average (n = 2)**	**Standard deviation (n = 2)**
Artemether	50	5.5	2.5	9.4	4.3
Artesunate	50	2.5	0.3	4.6	0.9
DHA	50	2.6	0.7	6.2	1.9
Atovaquone	400	2.1	0.3	5.5	0.3
Mefloquine	200	11.9	0.5	27.3	0.5
Piperaquine	200	46	9	68	3
Lumefantrine	200	34	4	99	13
Chloroquine	1,000	130	6	208	8
Pyrimethamine	5,000	2,799	52	4,080	248
Proguanil	200,000	5,677	617	18,840	5416

### Quality review of the *P. falciparum* blood-stage malaria cell banks

Following testing of the cell banks according to the criteria listed in Additional file 1, a quality review was undertaken to establish whether the *P. falciparum* cell banks were suitable for release and eventual administration to human volunteers in accordance with annex 13 of the PIC/S Guide and relevant guidelines. All documentation associated with the manufacture of the cell banks and the results of the testing outlined in Additional file 1 were reviewed and met with the established test criteria. The *P. falciparum* NF54, 3D7 and 7G8 cell banks were deemed to pass the required criteria for release.

## Discussion

The aim of the present study was to establish methodology for the production of GMP-grade, cultured malaria cell banks, as a new approach for producing material that can be used in human malaria blood-stage challenge studies to evaluate anti-malaria drugs and malaria vaccine candidates. Three blood-stage malaria cell banks, derived from three different starting materials (GMP-grade *P. falciparum* NF54 used to produce PfSPZ Vaccine [5,18,27] and PfSPZ Challenge [12-14], clinically isolated *P. falciparum* 3D7 [15] and research (non-GMP)-grade *P. falciparum* 7G8) were manufactured in cleanrooms suitable for the production of sterile products using screened blood components utilising robust standard operating procedures. The cell banks were subsequently tested according to specific criteria and as required by local guidelines of the Australian TGA, and the results evaluated to determine whether the cell banks could be released for subsequent use in clinical malaria studies.

All of the *in vivo* expanded cell banks were shown to contain viable parasites. The quantification of viable parasites (as was calculated for the *P. falciparum* 3D7 and 7G8 cell banks), together with the parasitaemia of the cells banks immediately prior to cryopreservation, can be used to calculate the volume of the cell bank suspension that is required to deliver a defined dose of viable parasites in clinical studies.

Sterility and endotoxin testing, performed using validated techniques, demonstrated that all cell banks were negative for bacterial contamination. Similarly, all three cell banks were tested to comply with applicable region-specific regulations and guidelines and were demonstrated to be free of contamination with mycoplasma and the donors of the erythrocytes and serum were shown to be negative for a broad panel of viral agents. One of the produced banks (*P. falciparum* 7G8) was tested by PCR for blood borne viruses and was negative for all tested agents. This gives confidence in the biosafety of the product itself i.e. that when administered to human volunteers they will not result in an unintended infection with any of the tested adventitious agents.

Establishing the *in vitro* susceptibility of the *P. falciparum* parasites in the cell banks to a panel of anti-malaria compounds is a critical step in ensuring that the cell banks can be safely administered to human volunteers. These data inform selection of the anti-malaria medication that will be administered to human volunteers who have received the cell bank parasites, to ensure complete resolution of the malaria infection. The *P. falciparum* NF54 parasites were susceptible to all drugs tested. Subjects infected with *P. falciparum* NF54 parasite have been successfully treated with chloroquine, atovaquone/proguanil (Malarone®) and with artemether/lumefantrine (known as Coartem® or Riamet®) [12,13,28,29], and all would be appropriate treatment. The *P. falciparum* 7G8 parasites exhibited resistance to a number of the drugs tested, restricting the treatment options. Only mefloquine (Lariam®) has been used to treat this parasite in clinical trials [29,30]. However, artemether/lumefantrine would undoubtedly be effective. The parasite has reduced susceptibility to proguanil as compared to *P. falciparum* NF54, but is highly susceptible to atovaquone, so atovaquone/proguanil may be effective also. The *P. falciparum* 3D7 parasites demonstrated susceptibility to all compounds tested, so all would be appropriate treatment.

One of the advantages of this culture-based approach to generating a *P. falciparum* malaria cell bank is the ability to select the parasite strain. Currently, few *P. falciparum* strains are available with which to evaluate the strain-transcending nature of the immune response induced by vaccine candidates, prior to larger and more expensive field-based trials in malaria-exposed individuals. Strain or allele-specific immune responses induced by sub-unit vaccine candidates may have contributed to the disappointing efficacy observed thus far, when vaccine candidates have been evaluated in a field setting. The selection of numerous, defined parasite strains for the early evaluation of vaccine candidates will ensure that only the most promising are channelled into the more expensive field-based trials for evaluation of efficacy.

An important aspect of this culture-based approach is the possibility to take research-grade material through to a final, GMP-grade product. Thus, the lack of access to GMP-grade starting material will not impose restrictions on the selection of parasite strains for a cultured cell bank. While the conversion of research-grade material to GMP-grade is an unexplored approach for clinical malaria research, there is precedence for this in clinical stem cell research [31]. It involves the transfer of research (non-GMP)-grade material to a cleanroom suitable for production of sterile products, where it is further cultured using defined, GMP-quality reagents according to robust standard operating procedures. Rigorous testing must be undertaken on the final product to determine that it is suitable for release and administration to humans and should comply with the current, local GMP requirements. This testing will ensure that there is no biological contamination and will exclude the possibility that adventitious agents may have been present in the research-grade material prior to culture in the cleanroom.

Following the successful *in vitro* characterization and release of these *P. falciparum* blood-stage malaria cell banks in these current studies, future work will entail the characterization of these cell banks *in vivo*. As a result of these processes, the necessary regulatory and ethical clearances have been obtained to examine the infectivity of these cell banks in malaria-naïve human volunteers, as has been previously undertaken for non-cultured *P. falciparum* and *P. vivax* cell banks that have been derived from malaria infected volunteers [9,15,16].

## Conclusions

The current study describes the first alternate methodology for the production of blood-stage malaria cell banks for use in controlled human malaria infections in CHMI studies. The ability to undertake these studies is critical for advancing the development of a malaria vaccine and novel anti-malaria drugs. Unlike existing published methods, this alternate method is culture-based and is not restricted by access to volunteers who have been deliberately infected with malaria or who contracted malaria infection following travel to/residence in a malaria endemic area.

## Additional files

Additional file 1: Table S1. Release criteria for *P. falciparum* blood-stage malaria cell banks. Description of data: Release criteria for *P. falciparum* blood-stage malaria cell banks. Additional file 2: Table S2.
*In vitro* anti-malaria activities (IC_50_ and IC_90_) of 10 compounds against the *P. falciparum* strain D6. Description of data: *In vitro* anti-malaria activities (IC_50_ and IC_90_) of 10 compounds against the *P. falciparum* strain D6. Additional file 3: Table S3.
*In vitro* anti-malaria activities (IC_50_) of 8 compounds against the *P. falciparum* strain 3D7.Description of data: *In vitro* anti-malaria activities (IC_50_) of 8 compounds against the *P. falciparum* strain 3D7.
