# Supplementary material for: Development of cultured Plasmodium falciparum blood-stage malaria cell banks for early phase in vivo clinical trial assessment of anti-malaria drugs and vaccines
Source: Malar J. 2015 Apr 7;14:143. doi: 10.1186/s12936-015-0663-x (PMC4392471; doi:10.1186/s12936-015-0663-x)
Supplement: Additional file 1: Table S1. — Release criteria for P. falciparum blood-stage malaria cell banks. Description of data: Release criteria for P. falciparum blood-stage malaria cell banks. [file 12936_2015_663_MOESM1_ESM.pdf]

Supplementary Table 1: Release criteria for *P. falciparum* blood-stage malaria cell banks.

Test Description	Specification	Service Provider
Parasite Stage	>75% ring stage parasites	Griffith University Q-Gen
% Parasitemia	>1% ring stage parasitemia	Griffith University Q-Gen
Viability	Viable parasites upon thawing	Griffith University QIMR Berghofer Medical Research Institute (QIMRB)
Sterility testing	Report no growth	Biotest Pathology QLD
Mycoplasma testing	Report not detected	ams Laboratories Q-Gen
Endotoxin testing	Report <1250EU/ml	ams Laboratories Q-Gen
Viral testing	Report not detected	Charles River
Drug sensitivity for anti-malarials	Report *in vitro* susceptibility to anti-malarial compounds as determined by IC_{50} and IC_{90} values.	Australian Army Malaria Institute
