# Supplementary material for: Development of cultured Plasmodium falciparum blood-stage malaria cell banks for early phase in vivo clinical trial assessment of anti-malaria drugs and vaccines
Source: Malar J. 2015 Apr 7;14:143. doi: 10.1186/s12936-015-0663-x (PMC4392471; doi:10.1186/s12936-015-0663-x)
Supplement: Additional file 2: Table S2. — In vitro anti-malaria activities (IC50 and IC90) of 10 compounds against the P. falciparum strain D6. Description of data: In vitro anti-malaria activities (IC50 and IC90) of 10 compounds against the P. falciparum strain D6. [file 12936_2015_663_MOESM2_ESM.pdf]

Supplementary Table 2: *In vitro* anti-malaria activities (IC_{50} and IC_{90}) of 10 compounds against the *P. falciparum* strain D6.

Compound	Maximal concentration tested (nM)	IC_{50} (nM)		IC_{90} (nM)	
		Average (n=2)	Std Deviation (n=2)	Average (n=2)	Std Deviation (n=2)
Artemether	50	4.7	0.7	7.8	0.6
Artesunate	50	3.4	0.1	5.6	0.2
Dihydroartemisinin	35	1.9	0.1	4.1	0.8
Atovaquone	25	0.10	0.01	0.8	0.2
Mefloquine	728	40	1	86	27
Piperaquine	200	23	2	34	9
Lumefantrine	800	108	12	356	38
Chloroquine	250	12.9	4.4	18.5	7.4
Pyrimethamine	50	0.59	0.04	0.95	0.05
Proguanil	200,000	312	41	1,246	640
