# Supplementary material for: Development of cultured Plasmodium falciparum blood-stage malaria cell banks for early phase in vivo clinical trial assessment of anti-malaria drugs and vaccines
Source: Malar J. 2015 Apr 7;14:143. doi: 10.1186/s12936-015-0663-x (PMC4392471; doi:10.1186/s12936-015-0663-x)
Supplement: Additional file 3: Table S3. — In vitro anti-malaria activities (IC50) of 8 compounds against the P. falciparum strain 3D7.Description of data: In vitro anti-malaria activities (IC50) of 8 compounds against the P. falciparum strain 3D7. [file 12936_2015_663_MOESM3_ESM.pdf]

Supplementary Table 3: *In vitro* anti-malaria activities (IC_{50}) of 8 compounds against the *P. falciparum* strain 3D7.

**Compound**	**IC_{50} (pmol/well)**
Amodiaquine	1.55
Artemisinin	0.029
Chloroquine	0.49
Dihydroartemisinin	0.27
Mefloquine	3.21
Pyronaridine	<0.122
Quinine	4.13
Sulphadoxine	4702.6
